# Aging Does Not Enhance Social Contagion Effect

**DOI:** 10.3389/fpsyg.2021.691229

**Published:** 2021-08-04

**Authors:** Susana Carnero-Sierra, Julio Menor

**Affiliations:** Área Psicología Básica, Psychology Department, University of Oviedo, Oviedo, Spain

**Keywords:** false memory, social contagion, memory illusions, aging, false recall

## Abstract

Studies on the social contagion of memory show that it is possible to create false memories from the wrong responses from other people without requiring their physical presence. The current study examined age differences between false memories via the modified social contagion paradigm. Twenty older and twenty younger adults were shown six household scenes and were exposed to the erroneous memory reports of an implied confederate who was not physically present. The presentation time of the scenes and the typicality of the contagion items were manipulated. The participants watched each scene individually and then took turns giving their recall responses with the responses belonging to a fictional participant provided by written cards. The results in a final individual recall test indicated a significant contagion effect in both groups of participants. Additionally, an effect of the typicality of the contagion items was observed, such that the more typical items produced more contagion than the less typical items. In relation to true recall, the older adults remembered significantly fewer items from the scenes than the younger ones and obtained a lower score in the word list subtest of the Weschler Memory Scale. Although the older group had an episodic memory deficit, they were not more susceptible to being affected by the wrong responses of other people than younger group.

## Introduction

The process of remembering occurs, in many cases, in a social context since one of the purposes of the act of remembering is to transmit information to other people. As different studies have shown (Roediger et al., 2001; Harris et al., 2017), during this process the person who shares the memory can transmit wrong information. The phenomenon involved in this situation is called social contagion of memory. Most of the studies on the social contagion of memory have focused on young adults, but there are less studies that examine how age influences the susceptibility to wrong information socially introduced (Meade et al., 2020).

Regarding the formation of false individual memories and aging, it has been consistently observed that older participants are more susceptible to accepting misinformation (see Schacter et al., 1997, who reviewed the source of memory failures in induced false memory procedures often found in memory tasks in older participants). Additionally, Devitt and Schacter (2016) concluded that several neurological changes related to false memories are associated with aging, increasing susceptibility to false memories. Specifically, Roediger and Geraci (2007) found that older adults were more susceptible to being affected by misinformation interference in the eyewitness paradigm from Loftus and Palmer (1974). Roediger and McDaniel (2007) reviewed the results of four additional experimental paradigms: the DRM paradigm (Roediger and McDermott, 1995), the categorized word list (Meade and Roediger, 2006), the misinformation paradigm (Loftus and Palmer, 1974), and the imagination inflation paradigm (Goff and Roediger, 1998), ultimately concluding that older adults are more susceptible to false memories than young adults.

However, differences in social false memory paradigms between younger and older participants are a matter of controversy. Meade and Roediger (2009) used a turn-taking collaborative procedure from the recall list of categorized words, describing that older participants have more possibilities to suffer from false recall than younger participants in the final recall test. However, Henkel and Rajaram (2011) observed no age differences between younger and older adults using a free-flowing procedure for collaborating. Gabbert et al. (2003) employed a video and a suggestive questionnaire to induce false memories in younger and older participants. They found no age differences in false memories, although it was observed that false recall in a posterior individual recall is increased when the possibility of prior discussion with other participants is given. The authors understood it as a result of the memory conformity effect. In a subsequent article, Gabbert et al. (2004) went on in depth about a procedure to determine that, in both younger and older, a social chatting with a same-age confederate is more effective for inducing false memories than a biased written narrative. Interestingly, results shown that older were even less susceptible than younger to commit false recall. The third study that explicitly tested age differences in social contagion of memory was carried out by Davis and Meade (2013), who employed the Roediger et al. (2001) paradigm to test false memories in both older and younger participants. No age differences were found, although an effect of confederate age was observed. In summary, the studies carried out so far indicate that the effect of social memory contagion is similar in younger and older adults.

Meade et al. (2020) suggests that social contagion protocols with physical confederates could minimize source monitoring errors which more likely occur in older than in younger. Expressions, emotions, and distinctiveness of recall of having a physical partner could enhance the distinctiveness of erroneous items recalled by the confederate from items actually presented. If this explanation is correct, then providing the wrong answers in written protocols would lead to reducing the distinctiveness of the source and increasing monitoring errors. Since source monitoring errors occur more frequently in older than in younger, the older adults would commit more false memory than the younger adults.

The effect of social contagion on memory induced by a written protocol, that simulates the responses of other people, has already been investigated in some studies. Roediger et al. (2001) established a procedure to induce false memories through a protocol of social contagion. Later, Meade and Roediger (2002) made a substantial variation in the procedure. In original experiments (Roediger et al., 2001) a second experimenter was used, pretending to be a participant who performed the required tasks alongside the real participant. Instead, Meade and Roediger (2002) replaced the false participant with a written protocol provided by the researcher. The cards presented to the real participant transmitted the same information as the confederate of the previous procedure. In this way, it was explained that responses written on the cards consisted of identical responses given by a subject previously involved in the same task. Thereby, the social component of the experiment was not eliminated. Results of Meade and Roediger (2002) indicated that this modified version of the procedure was just as strong at inducing false memories as the original procedure. Menor and Carnero (2013) confirmed that there were no differences between this non-face-to-face mode of contagion and the face-to-face one, observing similar contagion rates in both conditions from a young adult sample.

The present study aimed to analyze age differences in social contagion of memory by using a virtual confederate. It extends upon previous research of age differences in social contagion of memory, by using, as source of contagion, the virtual paradigm without physical presence of the confederate. The absence of a face, a voice, and other social factors involved in recalling with another person, would decrease source monitoring, which is especially problematic for older participants (Mitchell et al., 2003; Devitt and Schacter, 2016). Two stimulus presentation times (15 and 60 s) were used, and high expectative (objects expected to be in a scene) and low expectative (non-frequent objects about the thematic of the scenes) contagion items were introduced by virtual confederate. In relation to the presentation time of the scenes, it has been found that when the contagion items are introduced by a physical confederate, a shorter presentation time of the scenes increases the false memory in young adults. Thus, Roediger et al. (2001) and Menor and Carnero (2013) found that 15-s rates increased false memory compared to 60-s rates. However, using a written protocol as a source of contagion, Menor and Carnero (2013) did not observe differences between 15 and 60 s. It is possible that the older group was more affected by the shorter presentation time due to their episodic memory deficit, so that older adults would rely more on the responses of the written protocol when they have to remember the scenes.

The results obtained in older adults were compared with those obtained by the group of young adults in Menor and Carnero (2013). Due to a decreased source distinctiveness through the absence of a physical confederate, it was expected to find more social contagion in older than young adults. Additionally, it is expected to find an interaction between the presentation rate and the age group in such a way that the 15-s rate would cause greater social contagion than the 60-s rate in the group of older adults, but not in the young adults group. Lastly, the high-expectation contagion items would cause greater social contagion than the low-expectation items in both groups of participants as observed in other studies (Roediger et al., 2001; Meade and Roediger, 2002).

## Materials and Methods

### Participants

Participants consisted of twenty older adults from municipal social centers for seniors. Seventeen women and three men between the ages of 60 and 88 ($\bar{x}$ = 72.37, *SD* = 8.57) participated, with no specific diagnosed pathology. Older participants scored within the clinically normal range on the MMSE (Folstein et al., 1975; Spanish version: Blesa et al., 2001), $\bar{x}$ = 27.25, *SD* = 1.68. The Geriatric Depression Scale (Yesavage et al., 1982) revealed no signs of depression ($\bar{x}$ = 4.93, *SD* = 3.59). Furthermore, the mean score on the vocabulary subtest of WAIS (Wechsler, 1999) was 40.93, *SD* = 6.36. Performance in episodic memory was tested using the word list subtest of the Wechsler Memory Scale-III (Wechsler, 2004). The results are shown later in the results section. Older participants’ results were then compared with a sample of younger adults (20 undergraduate students between 21 and 34 years old, thirteen women and seven men) that experienced the same procedure of social contagion through a written protocol (Menor and Carnero, 2013). The young adults had more years of education ($\bar{x}$ = 15.1, *SD* = 0.24) than the older adults ($\bar{x}$ = 13.5, *SD* = 3.6), *t*(38) = 2.45, *p* = 0.01, and *d* = 0.63.

### Materials

Six photographs were used to portray six typical scenes of a house: a toolbox, a bathroom, a kitchen, a bedroom, a pantry, and a desk, each containing an average of 21.16 objects. The photographs were composed and made expressly for the investigation and each of the objects that appeared in them were selected with a previous investigation following the same procedure of Roediger et al. (2001). Photographs of these scenes were taken in a real context and with real objects. These same materials were previously used with young adult participants, proving to be able to generate false memories (Menor and Carnero, 2013). Twenty-one people between the ages of 18 and 67 who participated in this pilot study, cited ten objects that could be in those scenes. Objects cited by a minimum of ten people were considered high expectation, while objects cited only by one person were considered low expectation. In each scene, four objects were selected, two with high expectation and another two with low expectation, which would not appear in the photographs and which served as contagion items. To build each of the scenes, the rest of the high and low expectation objects were used.

To carry out the contagion phase, a protocol was developed that replaced the physical subject who functioned as a source of social contagion. This protocol was developed similar to the protocol of Meade and Roediger (2002). Each item of the contagion was written on a white paper card in capital letters, which was presented to the participants at the corresponding time in the recall phase, together with the protocol. The list of items used as contagion for each scene is reflected in Table 1. Two contagion items appeared for each contagion scene. A high expectancy contagion item always appeared in the fourth position of the protocol, and a low expectancy item was given in the sixth position, as Meade and Roediger (2002) did. The experimenter also had a main item and a reserve item available, to be able to present if the subject would mention, previously and spontaneously, the main contagion items (see Table 1).

**TABLE 1:** Contagion items: The main contagion items for each scene highlighted in bold.

	**High**		**Low**	
Toolbox	**Pliers**	Adjustable wrench	**Torch**	Silicone
Bathroom	**Bar of soap**	Sponge	**Razor**	Nail clippers
Kitchen	**Pan**	Sink	**Coffee maker**	Napkins
Bedroom	**Lamp**	Carpet	**Pillow**	Slippers
Pantry	**Milk**	Rice	**Dustpan**	Potatoes
Desk	**Pen tin**	Stapler	**Ruler**	Magazines

*The reserve items appear in the adjacent column. Original cards were written in Spanish, the native language of participants.*

### Design and Procedure

The experiment followed a mixed 2 × 2 × 2 × 2 design composed of four variables with two levels each: age (older-younger), scenes exposed to contagion (contagion – no contagion), expectation of the contagion items (high expectation – low expectation), and presentation time of each scene (15–60 s). Exposure to social contagion and expectation of the contagion items were within-subject variables. The time was classified as inter-subject variable, half subjects were randomly assigned to the 15-s condition and others to the 60-s condition. It may be noted that only three of the six scenes watched were exposed to contagion items by protocol. The other three scenes were accompanied by all veridical items through the written protocol, serving as the control condition. The dependent variable was recorded throughout the number of contagion items recalled in the individual free recall test, expressed in proportion over total contagion items exposed. The correct rate of recall in each scene was also measured, over the total items presented in each photograph.

The experiment was carried out in an isolated room. At first, a presentation containing a sequence of the six photographs was viewed on a computer. To half of the participants, the condition of presenting 15 s of each photograph was used. To the other half, the item exposition condition was 60 s. Immediately afterward, the participants were asked to perform a distracting task for 4 min consisting of simple addition and subtraction. Once this was done, the first recall test and key phase for contagion began. It was explained to the participants that these images had already been presented to other individuals, and their recall responses were collected on the cards that the researcher had. Thus, this first test consisted of remembering six items from each scene, but the real participant had to establish a series of turns between their real responses and the card false responses provided by the experimenter. Half of the subjects participating in the 15-s condition were assigned to Group 1 and the other half to Group 2. The same was true for the participants who watched the 60-s presentation. The difference between Groups 1 and 2 was how the contagion-induced scenes were counterbalanced. For Group 1, the scenes that contained the contagion items in the protocol were the toolbox, the kitchen, and the pantry, while for Group 2, the contagion scenes were the bathroom, the bedroom, and the desk. Collaborative recall of the rest of the scenes followed the same procedure, but items written in cards were all items presented in the photographs, without contagion items. The proportion of contagion items that appear by chance in the three no-contagion scenes, served as the control condition. The next phase consisted of an individual recall test, in which the participant had to name all the objects that he was able to remember for each scene and with a time limit of 2 min for each scene. When presenting the scenes, the order for the viewing phase remained the same throughout all the tests.

## Results

A mixed ANOVA with mentioned variables was done with status of contagion items and expectation items as within-subject factors, and time of presentation and age group as between-subject factors. To begin with, the counterbalance of contagion and non-contagion scenes was analyzed to rule out any differences depending on the features of the scene. For this, the total number of contagion items remembered by the participants in half of the scenes was compared with the participants who suffered the contagion in the others (Group 1 and Group 2). It is confirmed that the counterbalance did not influence the total contagion based on the scenes, *F*(1, 38) = 1.09, *p* = 0.301, and η*_*p*_^2^* = 0.03.

**FIGURE 1:**
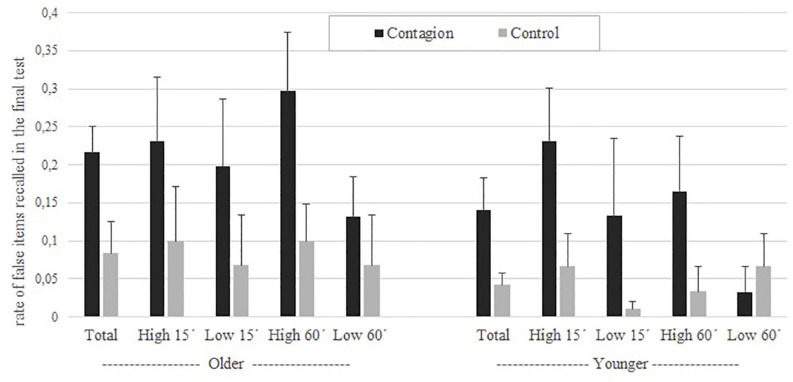
Rate of false items recalled between contagion and control conditions in both older and younger participants. Dark bars show contagion rate whereas light ones depict control performance. Corresponding standard errors are drawn over each bar.

Differences of contagion items remembered in the final individual test, between contagion and non-contagion scenes, indicated that participants remembered objects that did not actually appear in the scenes displayed but were suggested in the written protocol during the joint recall phase. A main effect of the total contagion was found, the mean proportion of false memory was higher in the contagion scenes than in the control scenes (see Figure 1), *F*(1, 36) = 12.49, *p* = 0.001, and η*_*p*_^2^* = 0.25. However, the age group factor was not significant, *F*(1, 36) = 2.20, *p* = 0.14, and η*_*p*_^2^* = 0.05, nor was its interaction with the contagion item, *F*(1, 36) = 0.22, *p* = 0.63, and η*_*p*_^2^* = 0.06. In fact, no other interactions were significant, *Fs*(1, 36) < 1.02, *ps* > 0.317, and η*_*p*_^2^* < 0.02. Expectation of the item was also found to be significant, *F*(1, 36) = 15.28, *p* < 0.001, and η*_*p*_^2^* = 0.29. Time for presentation was not significant, *F*(1, 36) = 0.42, *p* = 0.51, and η*_*p*_^2^* = 0.01.

In relation to veridical free-recall performance, the average of correctly recalled items belonging to the control scenes, in which no contagion items were introduced in the collaborative recall phase, was calculated. This was done in order to prevent the false memory from contaminating the veridical memory. An ANOVA was performed with exposition time (15 and 60 s) and age group (younger and older). This analysis revealed a significant main effect of age group, *F*(1, 36) = 11.98, *p* = 0.001, and η*_*p*_^2^* = 0.25, whereas older group participants recalled less items ($\bar{x}$ = 0.31) from scenes than younger group participants ($\bar{x}$ = 0.45). The exposition time of the scene was marginally significant, *F*(1, 36) = 3.56, *p* = 0.06, and η*_*p*_^2^* = 0.09, participants recalled more items when scenes were shown for 60 s each ($\bar{x}$ = 0.42) than when scenes were shown for 15 s each ($\bar{x}$ = 0.34). No interaction effect was observed, *F*(1, 36) = 0.009, and *p* = 0.92.

Regarding episodic memory performance, significant differences were found between younger and older participants in three measures of the word list subtest of Wechsler Memory Scale-III: first trial [younger $\bar{x}$ = 6.30, older $\bar{x}$ = 4.55, *t*(38) = 2.89, *p* = 0.006, and *d* = 0.92], total recall score after four trials [younger $\bar{x}$ = 36.35, older $\bar{x}$ = 26.55, *t*(38) = 5.02, *p* < 0.001, and *d* = 1.59], and learning slope [younger $\bar{x}$ = 5, older $\bar{x}$ = 3.75, *t*(38) = 2.10, *p* = 0.04, and *d* = 0.67]. No significant correlations were found between the total recall scores and social contagion scores in both younger (*r* = –0.23) and older participants (*r* = 0.30, *p*_*s*_ > 0.10).

## Discussion

The aim of this study was to verify whether the social memory contagion is greater in a group of older people than in a group of younger people, using a contagion protocol that does not require the physical presence of the confederate. The results showed that there were no significant differences between younger and older adults in the total contagion score. The presentation time for the scenes did not affect the false memory, however, expectation of the items showed a relevant effect, due to high expectation objects generating more contagion than low ones. The older group of participants did not show more expectation influence than the younger sample. Consequently, an effect of social contagion was obtained in both groups of participants, but social contagion was not superior in the older adults group. We had hypothesized more social contagion effect in older than younger participants due to the loss of distinctive clues in the written protocol. However, this did not occur. Therefore, written protocol seems to be as powerful as physical confederate, at least for generating the same level of source distinctiveness. Furthermore, these results extend those obtained by Davis and Meade (2013) who used a similar procedure with a physical confederate. In addition, although the group of older adults had a lower score in episodic memory than the group of younger adults, the magnitude of the effect of social contagion did not significantly correlate with episodic memory in either group.

Thus, it can be stated that the procedure for finding social contagion of memory through a written protocol seems adequate in older population. Therefore, it was enough for older participants to consider that the answers provided came from other participants in the same situation. In this regard, it is interesting to note that when the contagion items were presented in the protocol, the participants frequently claimed to remember those objects that did not appear in the photographs at any time.

The presentation time of the scenes did not significantly affect the social contagion of memory, nor did it interact with the age group. Unlike what was hypothesized, the shorter presentation time of the scenes did not increase the effect of social contagion in either of the groups. This result replicates that obtained by Menor and Carnero (2013), who found that the presentation time increased social contagion only when the contagion items were presented through a physical confederate, but not when it was virtual. It is important to note that the effects of presentation time are not consistent in the literature on false recall. Using other false memory paradigms (i.e., McDermott and Watson, 2001) an inverted *U*-shape function has been found, and Meade and Roediger (2002, exp. 2) did not found presentation time effects (5 vs 15 s per slide) using a physical confederate. Therefore, more research is needed to clarify the conditions under which the presentation time of scenes affects the paradigm of social contagion.

It is not infrequent to find diversity of results regarding the differences in tasks on false memories and the interference of false information in young and old adults. Roediger and Geraci (2007) explain these differences as the result of the different methodology used and the diversity of samples of older adults. However, as have been observed in Gabbert et al. (2003) following other procedure and Davis and Meade (2013) with the original protocol from Roediger et al. (2001), the results found in the present study supports the absence of age differences in a social contagion paradigm employing for the first time a written confederate protocol. Apparently, the social aspects included in contagion paradigms, even when using written protocols, contribute to neutralizing the enhanced false memories usually found in older people performing individual tasks.

The present study has some limitations that should be noted. The absence of differences between younger and older participants in social contagion could be due to the lack of power of the test to obtain these differences. A power sample analysis showed the need to improve the sample size used in this study to replicate this result, despite its similarity with Meade and Roediger (2002) experiments. Further research should also analyze individual differences among older adults since their cognitive performance is more variable than that of younger adults (Lindenberger and Oertzen, 2006). It is possible that the social memory contagion differs among older people due, for example, to variations in the ability to monitor memories and executive functioning (Colombel et al., 2016).

## Data Availability Statement

The raw data supporting the conclusions of this article will be made available by the authors, without undue reservation.

## Ethics Statement

The studies involving human participants were reviewed and approved by University of Oviedo. The patients/participants provided their written informed consent to participate in this study.

## Author Contributions

Both authors listed have made a substantial, direct and intellectual contribution to the work, and approved it for publication.

## Conflict of Interest

The authors declare that the research was conducted in the absence of any commercial or financial relationships that could be construed as a potential conflict of interest.

## Publisher’s Note

All claims expressed in this article are solely those of the authors and do not necessarily represent those of their affiliated organizations, or those of the publisher, the editors and the reviewers. Any product that may be evaluated in this article, or claim that may be made by its manufacturer, is not guaranteed or endorsed by the publisher.
